# Prostaglandin I_2_ upregulates the expression of anterior pharynx‐defective‐1α and anterior pharynx‐defective‐1β in amyloid precursor protein/presenilin 1 transgenic mice

**DOI:** 10.1111/acel.12495

**Published:** 2016-05-30

**Authors:** Pu Wang, Pei‐Pei Guan, Jing‐Wen Guo, Long‐Long Cao, Guo‐Biao Xu, Xin Yu, Yue Wang, Zhan‐You Wang

**Affiliations:** ^1^ College of Life and Health SciencesNortheastern UniversityShenyang110819China

**Keywords:** β‐amyloid protein, anterior pharynx‐defective‐1α/1β, APP/PS1, cyclooxygenase‐2, prostaglandin I_2_

## Abstract

Cyclooxygenase‐2 (COX‐2) has been recently identified to be involved in the pathogenesis of Alzheimer's disease (AD). Yet, the role of an important COX‐2 metabolic product, prostaglandin (PG) I_2_, in the pathogenesis of AD remains unknown. Using human‐ and mouse‐derived neuronal cells as well as amyloid precursor protein/presenilin 1 (APP/PS1) transgenic mice as model systems, we elucidated the mechanism of anterior pharynx‐defective (APH)‐1α and pharynx‐defective‐1β induction. In particular, we found that PGI_2_ production increased during the course of AD development. Then, PGI_2_ accumulation in neuronal cells activates PKA/CREB and JNK/c‐Jun signaling pathways by phosphorylation, which results in APH‐1α/1β expression. As PGI_2_ is an important metabolic by‐product of COX‐2, its suppression by NS398 treatment decreases the expression of APH‐1α/1β in neuronal cells and APP/PS1 mice. More importantly, β‐amyloid protein (Aβ) oligomers in the cerebrospinal fluid (CSF) of APP/PS1 mice are critical for stimulating the expression of APH‐1α/1β, which was blocked by NS398 incubation. Finally, the induction of APH‐1α/1β was confirmed in the brains of patients with AD. Thus, these findings not only provide novel insights into the mechanism of PGI_2_‐induced AD progression but also are instrumental for improving clinical therapies to combat AD.

## Introduction

Alzheimer's diseases (AD) is the most common cause of dementia in aged people and is characterized clinically by cognitive decline and pathologically by the accumulation of amyloid β‐protein (Aβ) and hyperphosphorylation of tau in the brain (Hoshino *et al*., 2007; Arnaud *et al*., 2009). Here, increases in the expression of several potentially toxic secretases, including BACE‐1, presenilin 1/2 (PS1/2), anterior pharynx‐defective (APH)**‐**1α/1β, nicastrin, and PEN2, result in the formation of Aβ plaques, synapse dysfunction or loss, neuronal loss, and diffuse brain atrophy, thereby leading to the decline of cognitive abilities (De Strooper, 2003). The γ‐secretases, such as APH‐1α and APH‐1β, are required for notch pathway signaling, for γ‐secretase cleavage of β‐APP, and for Aβ protein accumulation in *C. elegans* (Francis *et al*., 2002). Indeed, APH‐1 usually interacts with PEN‐2, nicastrin, and PS to generate an active form of the γ‐secretase complex, which is responsible for the cleavage of β‐APP and the deposition of Aβ (De Strooper, 2003). Once APH‐1 was found in *C. elegans* (Francis *et al*., 2002), the APH‐1 complex was then confirmed in several experimental models (Gu *et al*., 2003; Luo *et al*., 2003; Hansson *et al*., 2004). However, the regulatory mechanism of APH‐1α and APH‐1β are often overlooked during the course of AD progression.

To reveal the possible mechanism of APH‐1α and APH‐1β regulation, it is necessary to identify the molecular pathways that are responsible for the deposition of Aβ. Epidemiological and clinical data suggest that nonsteroidal anti‐inflammatory drugs (NSAIDs) are beneficial in the treatment and prevention of AD (Imbimbo *et al*., 2010). The protective effects of NSAIDs in AD are due to their anti‐inflammatory properties that inhibit cyclooxygenase‐2 (COX‐2) (McGeer, 2000; van Gool *et al*., 2003). As an important factor in inflammatory reactions of peripheral tissues, COX‐2 has a potential role in the pathogenesis of AD. This has been populously investigated through studies of its metabolic products, the prostaglandins (PGs), including PGE_2_, PGD_2_ [and its dehydration end product 15‐deoxy‐Δ^12,14^‐PGJ_2_ (15d‐PGJ_2_)], PGI_2_, PGF_2α,_ and TXA_2_ (Akarasereenont *et al*., 1999). For example, PGE_2_ treatment increases the ratio of Aβ_1–42_/Aβ_1–40_ production in SH‐SY5Y cells and APP23 transgenic mice (Hoshino *et al*., 2007, 2009). In line with these observations, PGE_2_ was further verified to stimulate the production of Aβ_1–42_ alone in C57BL/6 mice (Echeverria *et al*., 2005). In addition to PGE_2_, PGD_2_ shows positive effects on stimulating the production of Aβ_1–42_ in primary mouse microglia and neuronal cells (Bate *et al*., 2006). As expected, 15d‐PGJ_2_ is also able to increase fibrillar Aβ in rat cortical neurons (Takata *et al*., 2003; Yamamoto *et al*., 2011) while PGF_2α_ has been shown to be involved in Aβ production in microglia cells (Zhuang *et al*., 2013). However, the effects of PGI_2_ on the production of Aβ are not well studied.

In this study, an intracellular signaling pathway by which PGI_2_ regulates the expression of APH‐1α/1β has been proposed to contribute to the deposition of Aβ. Specifically, PGI_2_ treatment increases the expression of APH‐1α/1β via the PKA/CREB and JNK/c‐Jun activation pathways in SH‐SY5Y cells, which facilitates the synthesis of Aβ in neuron cells. More importantly, in AD, the Aβ oligomers were localized to the CSF microenvironment, which contributes to the expression of APH‐1α/1β. Reconstructing the signaling network that regulates PGI_2_‐mediated APH‐1α/1β expression in neuron cells will facilitate the development of strategies to combat AD.

## Results

### APH‐1α/1β is highly induced in APP/PS1 transgenic mice

**Figure 1:**
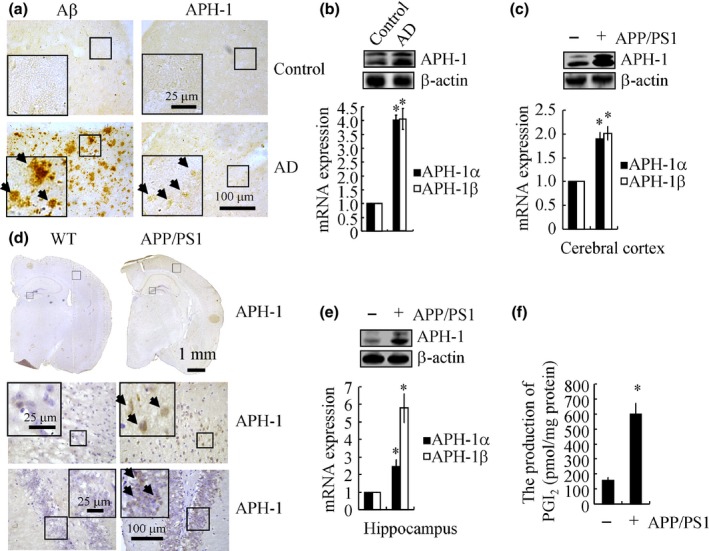
The expression of APH‐1α/1β is markedly increased in patients with AD and APP/PS1 transgenic mice at 6 months of age when compared with control subjects. The tissue blocks of human brains of AD were collected by the New York Brain Bank at Columbia University and Fengtian Hospital of China. Free‐floating slices (40 μm) were prepared using a cryostat (a, b). In selected experiments, the brains of APP/PS1 transgenic mice at 6 months of age were collected following anesthesia and perfusion (c–f). The immunoreactivity of APH‐1 or Aβ_1–42_ was determined by IHC using an anti‐APH‐1 or Aβ_1–42_ antibody (a, d). APH‐1α/1β mRNA and protein levels were determined by qRT–PCR and immunoblotting (*n* = 3 for human sample, *n* = 6 for mouse samples) (b, c, e). The production of PGI_2_ in the brains of APP/PS1 transgenic or of WT mice was determined by PGI_2_ determination kits (f). The data represent the means ± S. E. of at least three independent experiments. *P < 0.05 with respect to the WT or normal human controls.

Due to previous reports of a pivotal role of APH‐1α/1β in the pathogenesis of AD (Mrak & Griffin, 2001; De Strooper, 2003), we evaluated the expression levels of APH‐1α/1β in AD brains. As shown in Fig. 1a, the immunoreactivity of APH‐1α/1β and Aβ was highly induced at the patients with AD. In accordance with this observation, the mRNA and protein expression levels of APH‐1α/1β were elevated at the patients with AD (Fig. 1b). Due to the limited accessibility of human AD samples, we performed similar experiments in APP/PS1 mice. The results demonstrated that APH‐1α/1β immunostaining was markedly increased in the cerebral cortex as well as in the dentate gyrus region of the hippocampus of APP/PS1 transgenic mice at 6 months of age, when compared to the WT C57BL/6 mice (Fig. 1d). In accordance with our immunostaining observations, the mRNA and protein expression of APH‐1α/1β was upregulated in the cerebral cortex and hippocampus of APP/PS1 mice (Fig. 1c,e). When considered together, these results clearly demonstrate that APH‐1α/1β levels were increased in APP/PS1 transgenic mice. These data agree with previous reports (De Strooper, 2003), suggesting that APH‐1α/1β are possibly involved in aggravating AD.

### Elevation of PGI_2_ accelerates the synthesis of APH‐1α/1β in APP/PS1 transgenic mice

**Figure 2:**
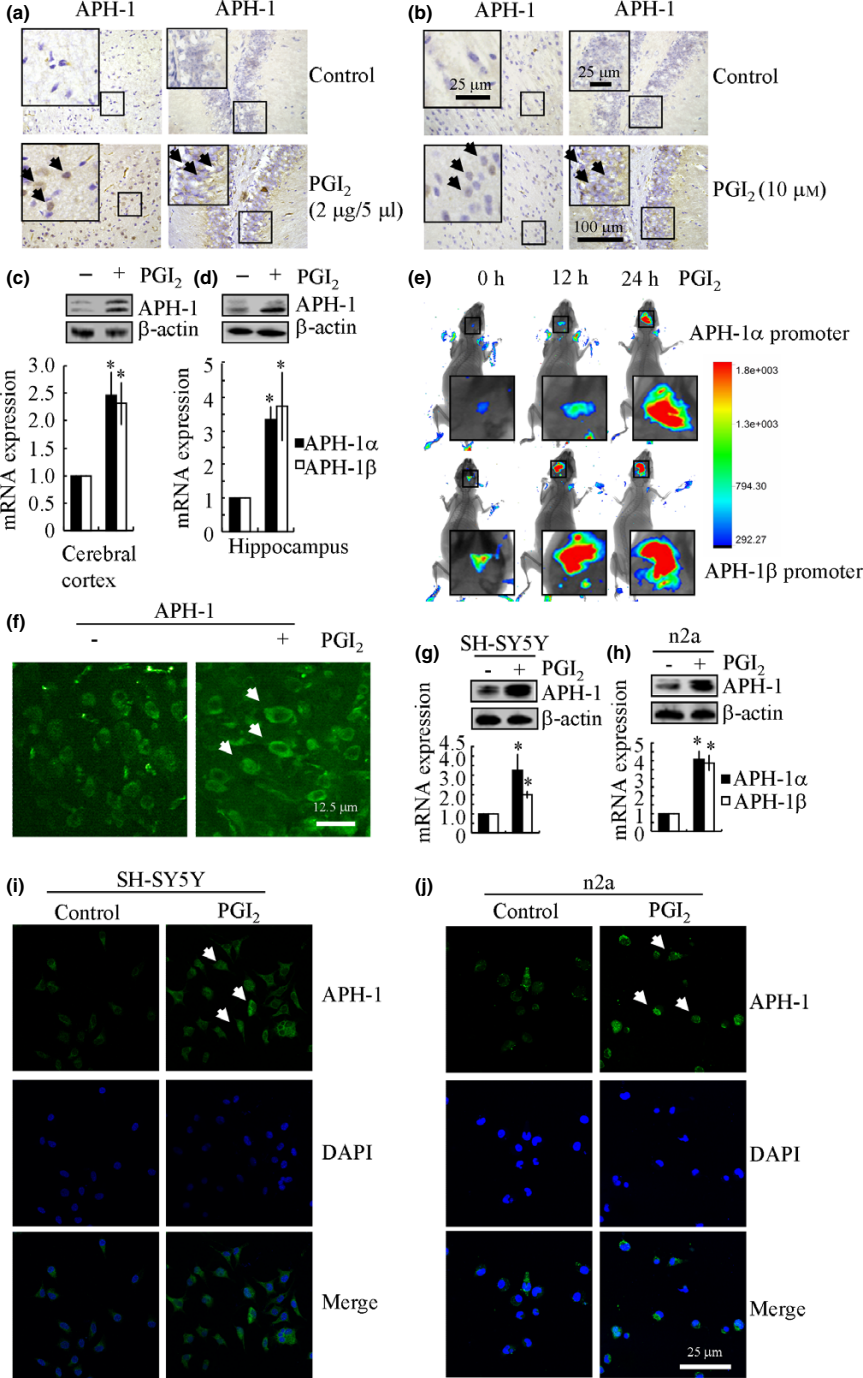
Involvement of PGI_2_ in inducing the expression of APH‐1α/1β. (a, c, d) The WT C57BL/6 mice at 6 months of age were injected (i.c.v.) with PGI_2_ (2 μg/5 μL), and the brains were then collected and sectioned after 24 h. The immunoreactivity of APH‐1 was determined by immunohistochemistry using an anti‐APH‐1 antibody (a). APH‐1α/1β mRNA and protein levels were determined by qRT–PCR and Western blots, respectively (*n* = 8) (c, d). (b) The brains of WT C57BL/6 mice at 6 months of age were harvested and freshly sectioned (400 μm) before treatment with PGI_2_ (10 μm) for 24 h. The slices were immunostained with APH‐1 antibody. (e) PGI_2_ (2 μg/5 μL) or vehicle (PBS) was injected (i.c.v.) into one side of a cerebral ventricle in the absence or presence of preseeded n2a cells that were transfected with APH‐1α/β promoters in the other side of cerebral ventricle (*n* = 6). Luciferase activities from the different groups of mice were measured using a live animal imaging system. (f) The left cerebral ventricle was injected with PGI_2_ (2 μg/5 μL) or vehicle (PBS) solution before staining with an APH‐1 antibody and scanning using two‐photon microscopy (*n* = 6). (g–j) SH‐SY5Y or n2a cells were treated with PGI_2_ (10 μm) for 48 h. APH‐1α/1β mRNA and protein levels were determined by qRT–PCR and Western blots, respectively (g, h). The immunofluorescence of APH‐1 was determined by immunohistochemistry using a primary rabbit APH‐1 antibody and secondary Alexa Fluor 488‐labeled goat anti‐rabbit IgG (i, j). The data represent the means ± SE of three independent experiments. *P < 0.05 with respect to the vehicle‐treated control.

We next sought to elucidate the mechanism by which APH‐1α/1β are upregulated in an AD mouse model. Because evidence suggests that PGI_2_ is a potential mediator of neuroinflammation (Ford‐Hutchinson *et al*., 1978; Honda *et al*., 2005; Pulichino *et al*., 2006), we sought to determine the concentration of PGI_2_ in APP/PS1 transgenic mice at 6 months of age. As shown in Fig. 1f, the synthesis of PGI_2_ was markedly increased in APP/PS1 transgenic mice. To know the roles of PGI_2_ in AD development, we first screened the effects of PGI_2_ on the expression of α‐, β‐ or γ‐secretases. The results demonstrated that PGI_2_ treatment concurrently downregulated the expression of ADAM‐10 and upregulated the expression of BACE‐1, PS2, and APH‐1α/1β in n2a cells (Table 1). To further understand the possible role of PGI_2_ in AD, we then injected (i.c.v.) PGI_2_ (2 μg/5 μL) or incubated brain slices from WT C57BL/6 mice with PGI_2_ (10 μm) for 24 h. IHC staining indicated that APH‐1α/1β expression was induced in both the cerebral cortex and the DG region of APP/PS1 transgenic mice (Fig. 2a,b). In addition, these observations were verified by qRT–PCR and Western blots (Fig. 2c,d). To identify the role of PGI_2_ in inducing the expression of APH‐1α/1β, we conducted live animal and two‐photon imaging experiments, as described in the ‘Materials and Methods’. The results revealed that PGI_2_ (2 μg/5 μL) injection (i.c.v.) into the ventricles of WT C57BL/6 mice increased the luciferase activity of the APH‐1α/1β promoters at 12 h following injection. This activity was maximal at 24 h following injection (Fig. 2e). Of note, PBS (−) injection (i.c.v.) does not alter the activity of APH‐1α and APH‐1β promoters (data not shown). Two‐photon imaging results reinforced the notion that PGI_2_ (2 μg/5 μL) injection (i.c.v.) increased the immunofluorescence of APH‐1α/1β in the cerebral cortex of WT C57BL/6 mice (Fig. 2f). Accordingly, we then treated the SH‐SY5Y and n2a neuronal cells with PGI_2_ (10 μm) for 48 h. Our data demonstrated that PGI_2_ treatment clearly increases the expression of APH‐1α/1β in human or mouse neurons (Fig. 2g,h). These results were supported by the immunostaining experiments, and the images obtained using Leica confocal microscopy (Fig. 2i,j). Collectively, our data clearly support the fact that PGI_2_ elevation increases the synthesis of APH‐1α/1β *in vitro* and *in vivo*.

**Table 1:** The effects of PGI_2_ on the expression of α‐, β‐, or γ‐secretases in n2a cells

Gene Name	Control	PGI_2_ (10 μm)
ADAM‐10	1	0.59
BACE‐1	1	4.59
PS1	1	0.99
PS2	1	1.92
APH‐lα	1	3.25
APH‐lβ	1	1.99
Nicastrin	1	0.94
PEN2	1	1.08

### PGI_2_ inhibition impaired the expression of APH‐1α/1β following intranasal administration of NS398 in APP/PS1 transgenic mice

**Figure 3:**
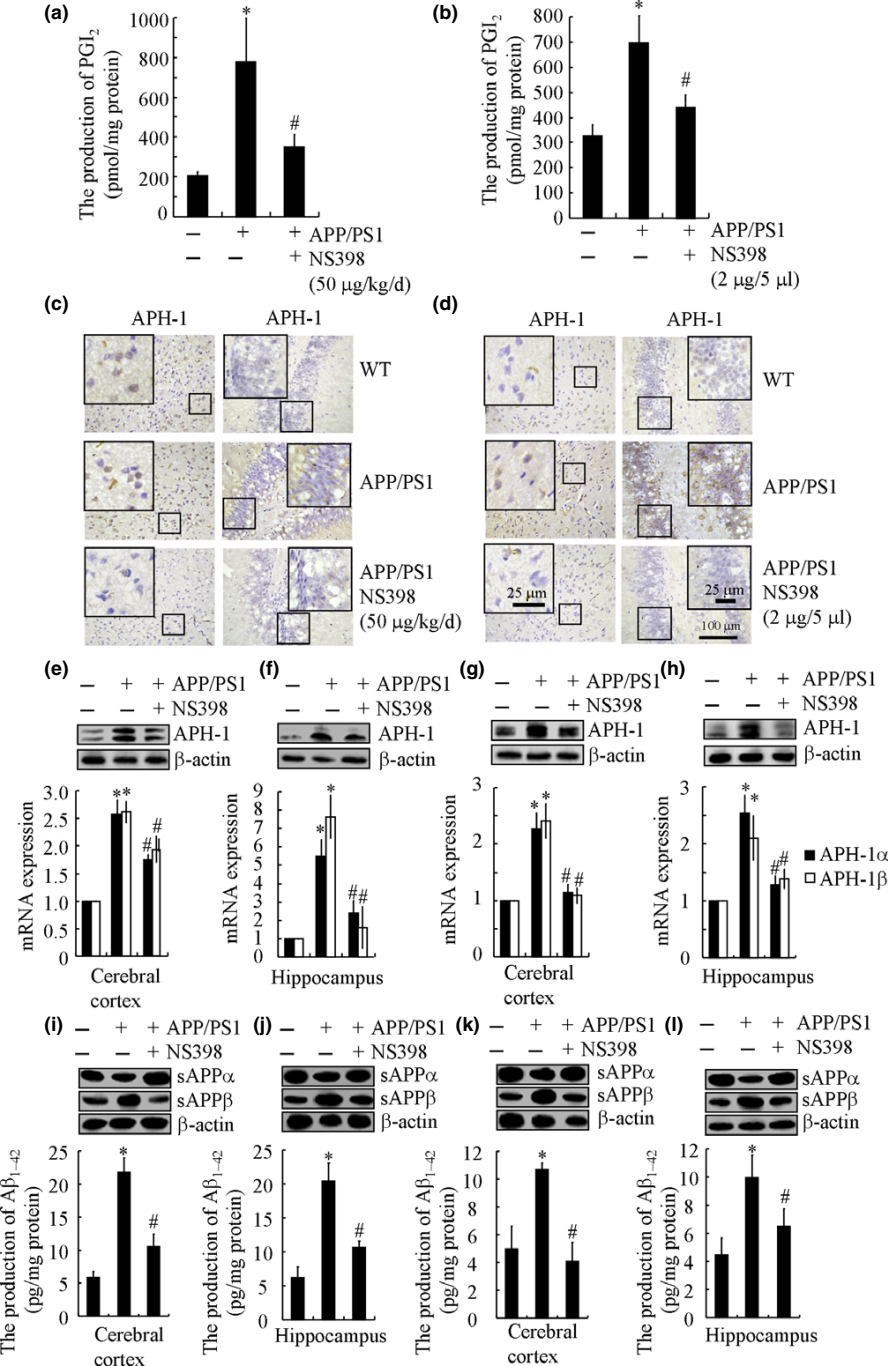
NS398 administration decreases the expression of APH‐1α/1β via inhibiting the production of PGI_2_ in APP/PS1 transgenic mice. The APP/PS1 transgenic mice at 1 month of age received NS398 (50 μg kg^−1^ day^−1^) for 5 months before harvesting brain slices (*n* = 3) (a, c, e, f, i, j). In selected experiments, the APP/PS1 transgenic mice at the 6 months of age were injected (i.c.v.) with NS398 (2 μg/5 μL). The brains were then collected and sectioned after 24 h (*n* = 8) (b, d, g, h, k, l). The production of PGI_2_ was determined by PGI_2_ enzyme immunoassay kits (a, b). The immunoreactivity of APH‐1 was determined by immunohistochemistry using an anti‐APH‐1 antibody (c, d). APH‐1α/1β mRNA and protein expression levels were determined by qRT–PCR and Western blots, respectively (e–h). The production of sAPPα and sAPPβ was determined by Western blots (i–l). The production of Aβ_1–42_ was determined by Aβ_1–42_ ELISA kits (i‐l). The data represent the means ± SE of three independent experiments. *P < 0.05 with respect to WT mice. ^#^ *P < 0.05* compared to APP/PS1 transgenic mice.

Because PGI_2_ is an important metabolic product of COX‐2, we treated APP/PS1 transgenic mice with a COX‐2‐specific inhibitor, NS398 (50 μg kg^−1^ day^−1^), for 5 months. Our results revealed that PGI_2_ was significantly suppressed by NS398 (50 μg kg^−1^ day^−1^) administration in APP/PS1 transgenic mice (Fig. 3a). More interestingly, the inhibition of PGI_2_ by NS398 resulted in a decrease in the levels of APH‐1α/1β in APP/PS1 transgenic mice by IHC staining (Fig. 3c). Similar results were verified by qRT–PCR or Western blot experiments (Fig. 3e,f). Of note, NS398 treatment does not affect the body weight of mice and induce wound healing to the mice as previously indicated (Wang *et al*., 2011a). We then injected APP/PS1 transgenic mice with NS398 (2 μg/5 μL) for 24 h. The results demonstrated that the injection (i.c.v.) of NS398 (2 μg/5 μL) shows a suppressive effect on the production of PGI_2_ in APP/PS1 transgenic mice (Fig. 3b). Of note, our results reinforce the hypothesis that NS398 treatment (2 μg/5 μL) suppressed the expression of APH‐1α/1β (Fig. 3d,g,h) by inhibiting the production of PGI_2_. In addition, the production of sAPPα was restored to the control level (i.e. the basal level of WT mice), whereas the increased production of sAPPβ was attenuated to the basal level of WT mice following intranasal administration of NS398 treatment for 5 months in APP/PS1 mice (Fig. 3i,j). Intranasal administration of NS398 also decreased the production of Aβ_1–42_ in APP/PS1 mice (Fig. 3i,j), which potentially decelerates the pathogenesis of AD. To further verify the role of NS398 in suppressing the expression of APH‐1α/1β, we performed intracerebroventricular injections of the inhibitor. Similar to nasal administration, NS398 injection (i.c.v., 2 μg/5 μL) reversed the concurrent downregulation of sAPPα and upregulation of sAPPβ in APP/PS1 mice (Fig. 3k,l). Therefore, our results concretely support the hypothesis that NS398 has the ability to suppress the expression of APH‐1α/1β by decreasing the production of PGI_2_ in APP/PS1 transgenic mice.

### Critical role of PKA/CREB and JNK/c‐Jun signaling pathways in mediating PGI_2_‐induced APH‐1α/1β expression in n2a cells

**Figure 4:**
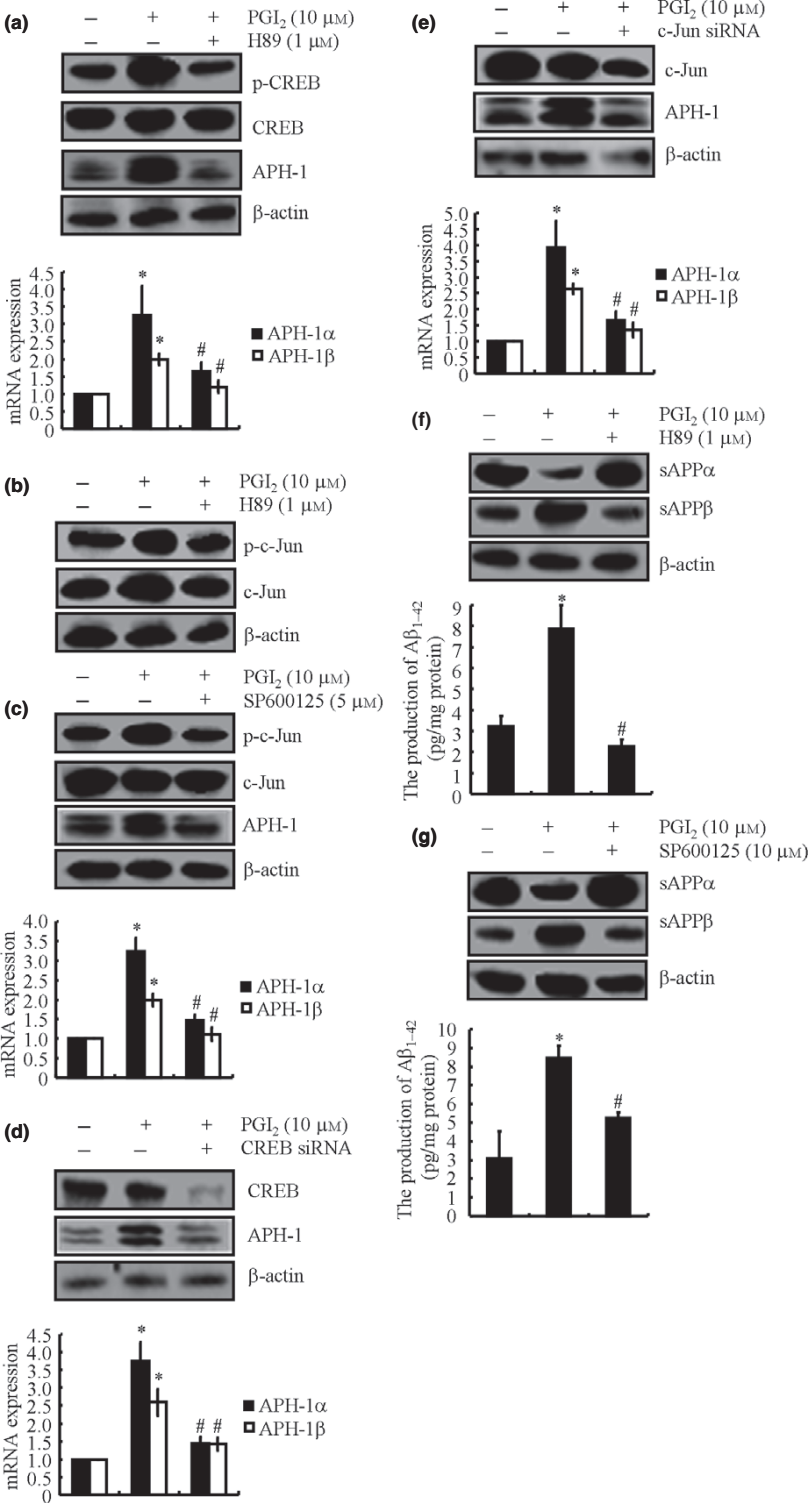
PGI_2_ elevation stimulates the expression of APH‐1α/1β via the PKA/CREB and JNK/c‐Jun signaling pathways in cultured neuronal cells. n2a cells were treated with PGI_2_ (10 μm) in the absence or presence of H89 (1 μm) (a, b, f) or SP600125 (5 μm) (c, g) cells for 48 h. In distinct experiments, n2a cells were transfected with CREB (d) or c‐Jun siRNA (e) before treating the cells with PGI_2_ (10 μm) for 48 h. APH‐1α/1β mRNA and protein levels were determined by qRT–PCR and Western blots, respectively (a, c–e). Phosphorylated CREB and c‐Jun as well as total CREB and c‐Jun were detected by immunoblotting using specific antibodies (a–e). The production of sAPPα and sAPPβ was determined by Western blots (f, g). The production of Aβ_1–42_ was determined by Aβ_1–42_ ELISA kits (f, g). The data represent the means ± SE of three independent experiments. *P < 0.05 with respect to the vehicle‐treated or vector‐transfected control. ^#^ *P < 0.05* compared to PGI_2_‐treated alone.

We next aimed to elucidate the signaling pathways of APH‐1α/1β synthesis in PGI_2_‐treated n2a cells. First, 48 h of PGI_2_ (10 μm) treatment activated PKA/CREB and JNK/c‐Jun signaling pathways by the phosphorylation of CREB and c‐Jun (Fig. 4a–c), which resulted in the synthesis of APH‐1α and APH‐1β in n2a cells (Fig. 4a,c). To further elucidate the role of PKA/CREB and JNK/c‐Jun signaling pathways in regulating the expression of APH‐1α/1β, we treated n2a cells with the PKA pharmacological inhibitor H89 (1 μm) or JNK‐specific inhibitor SP600125 (10 μm). Treatment of n2a cells with H89 (1 μm) or SP600125 (10 μm) not only suppressed the phosphorylation of CREB and c‐Jun (Fig. 4a–c) but also reversed the synthesis of APH‐1α/1β in PGI_2_‐treated n2a cells (Fig. 4a,c).

To verify these observations and to account for the nonspecificity of the pharmacological inhibitors, we transfected n2a cells with siRNAs that were specific for interfering with the expression of CREB or c‐Jun prior to incubating them with PGI_2_ (10 μm). As shown in Fig. 4d and e, CREB and c‐Jun knockdown efficiently decreased the protein levels of CREB and c‐Jun. As a consequence, the knockdown of CREB or c‐Jun inhibited the effects of PGI_2_ on inducing the synthesis of APH‐1α/1β in n2a cells (Fig. 4d,e). In addition, inhibiting the signaling pathways of PKA/CREB and JNK/c‐Jun concurrently results in the restoration of the production of sAPPα and a decrease in the production of sAPPβ to the basal level in PGI_2_‐treated n2a cells (Fig. 4f,g). More importantly, inhibiting the activity of the PKA/CREB or the JNK/c‐Jun signaling pathways resulted in the attenuation of Aβ_1–42_ formation in PGI_2_‐activated n2a cells (Fig. 4f,g). Therefore, these observations support the hypothesis that PKA/CREB and JNK/c‐Jun signaling pathways are important in mediating PGI_2_‐induced APH‐1α/1β expression, which results in Aβ_1–42_ deposition in neuron cells.

### Aβ oligomers in the CSF of APP/PS1 mice have the ability to stimulate the expression of APH‐1α/1β

**Figure 5:**
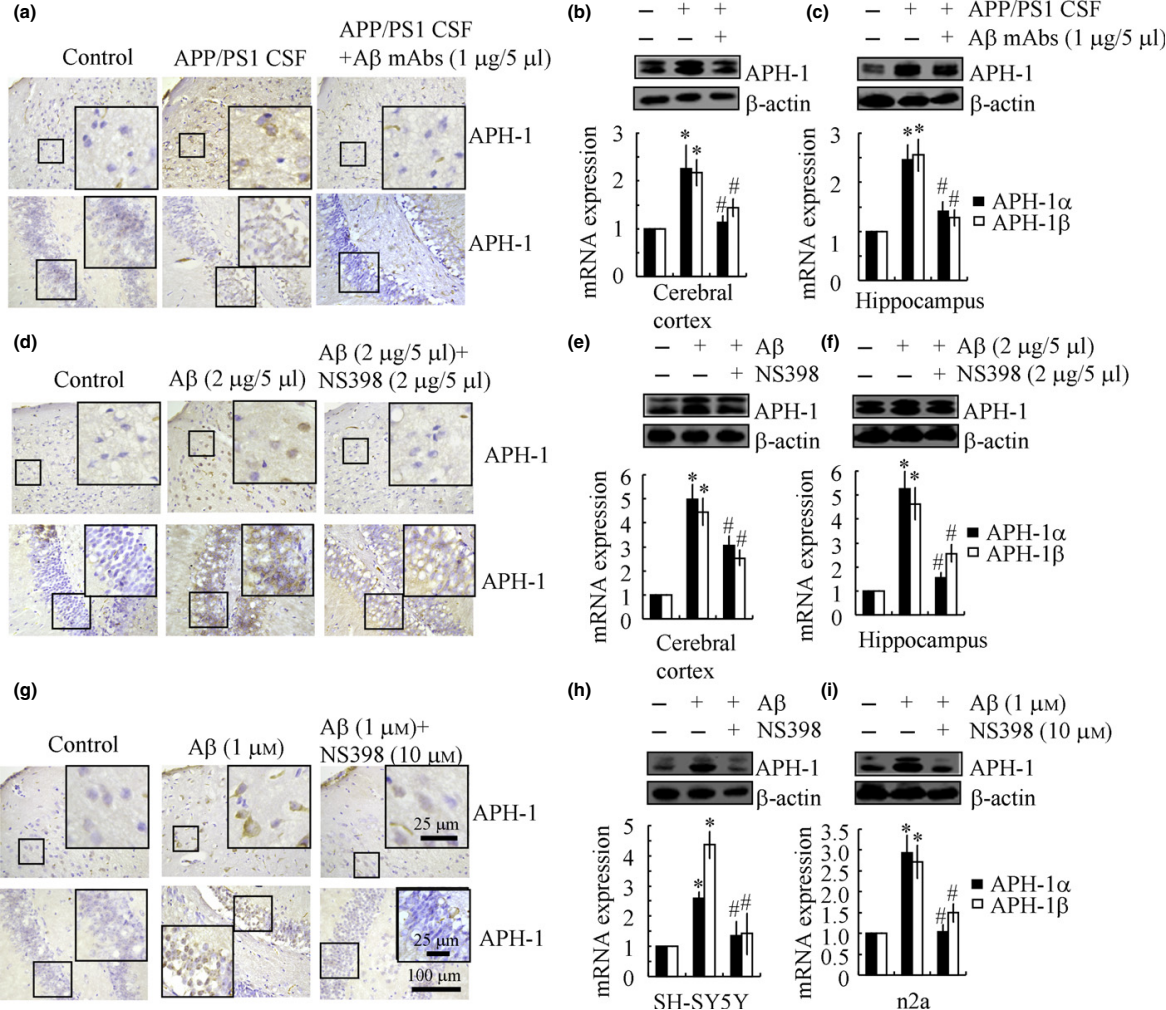
NS398 treatment diminished the effects of Aβ oligomers on inducing the expression of APH‐1α/1β. Cerebrospinal fluid (CSF) was obtained from APP/PS1 transgenic mice, which was then injected (i.c.v.), in the absence or presence of Aβ antibody (1 μg/5 μL), into C57BL/6 mice for 2 weeks before sacrifice (a–c). In selected experiments, the WT C57BL/6 mice at 6 months of age were injected (i.c.v.) with Aβ oligomers (2 μg/5 μL) in the absence or presence of NS398 (2 μg/5 μL). The brains were then collected and sectioned after 24 h (d–f). In separate experiments, the brains of WT C57BL/6 mice at 6 months of age were harvested and freshly sectioned (400 μm) before treatment with Aβ (1 μμ) in the absence or presence of NS398 (10 μm) for 24 h (g). In distinct experiments, SH‐SY5Y or n2a cells were treated with Aβ (1 μm) in the absence or presence of NS398 (10 μm) for 24 h (h, i). The immunoreactivity of APH‐1 was determined by IHC using an anti‐APH‐1 antibody (a, d, g). APH‐1α/1β mRNA and protein expression was determined by qRT–PCR and Western blots, respectively (*n* = 8) (b, c, e, f, h, i). The data represent the means ± SE of three independent experiments. *P < 0.05 with respect to vehicle‐treated controls. ^#^ *P < 0.05* compared to APP/PS1 CSF‐ or Aβ‐treated alone.

Because NS398 incubation decreases the expression of APH‐1α/1β and Aβ_1–42_ deposition by decreasing the production of PGI_2_ *in vitro* and *in vivo*, we next examined the potential contribution of Aβ_1–42_ to the pathogenesis of AD. We conducted experiments to determine whether Aβ_1–42_ is confined to microenvironments that are related to AD in APP/PS1 transgenic mice. In brief, the cerebrospinal fluid (CSF) of APP/PS1 transgenic mice was injected into WT C57BL/6 mice in the absence or presence of Aβ antibody for 2 weeks prior to sacrifice. When compared to control animals, the expression of APH‐1α/1β in both the cerebral cortex and hippocampus was markedly increased by the injection (i.c.v.) of APP/PS1 CSF (Fig. 5a–c). In addition, the elevated induction of APH‐1α/1β was suppressed by the injection (i.c.v.) of the Aβ antibody (1 μg/5 μL) (Fig. 5a–c). Therefore, these observations demonstrate that the confinement of the secreted form of Aβ_1–42_ to AD‐related microenvironments might induce the expression of APH‐1α/1β in a PGI_2_‐dependent manner.

To confirm these observations, we performed experiments to directly evaluate the involvement of Aβ_1–42_ oligomers in the progression of AD. Aβ oligomer (2 μg/5 μL) injection (i.c.v.) clearly increases the expression of APH‐1α/1β in both the cerebral cortex and hippocampus of WT C57BL/6 mice (Fig. 5d–f). The upregulation of APH‐1α/1β was further suppressed by NS398 (2 μg/5 μL) injection (i.c.v.) in Aβ_1–42_‐stimulated C57BL/6 mice (Fig. 5d–f). These *in vivo* observations were reinforced by *in vitro* experiments that demonstrated that Aβ (1 μm) treatment stimulates the expression of APH‐1α/1β in neuron cells by organotypic slice or cell culture (Fig. 5g–i). Thus, Aβ_1–42_ oligomers are critical for worsening AD.

## Discussion

**Figure 6:**
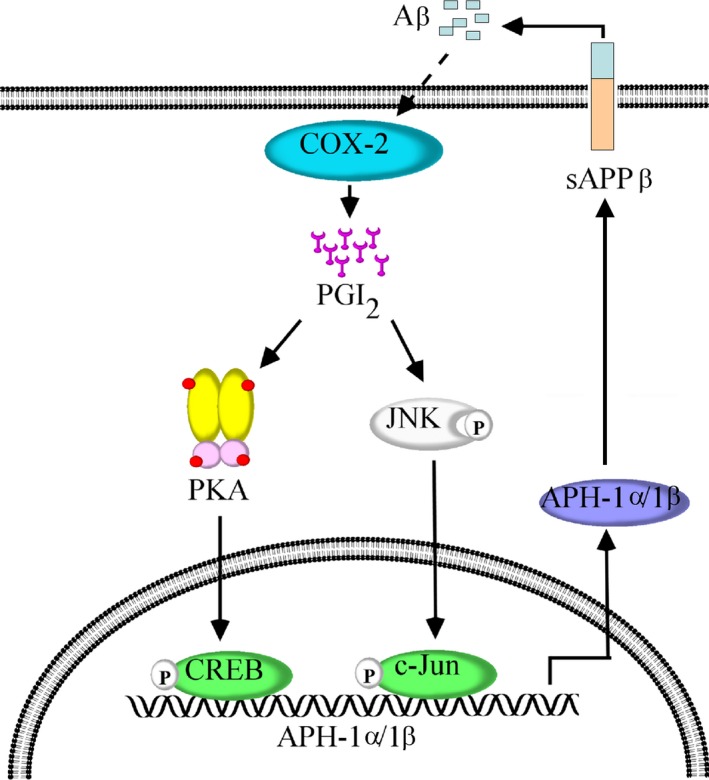
Proposed cascade of the signaling events regulating the pathogenesis of AD by PGI_2_. In detail, elevated levels of PGI_2_ in APP/PS1 transgenic mice will enhance the expression of APP‐1α/1β via the PKA/CREB and JNK/c‐Jun pathways in neuron cells of APP/PS1 transgenic mice, which in turn aggravates the pathogenesis of AD. Moreover, the highly secreted Aβ oligomers from neuron cells are able to reciprocally regulate the expression of APH‐1α/1β, which further aggravate the pathogenesis of AD *in vivo*. PGI_2_, a metabolic product of COX‐2, inhibition by NS398 administration reversed the effects of APP/PS1 overexpression in stimulating the expression of APH‐1α/1β, which potentially contributes to improvement in study ability and decline in cognitive ability in APP/PS1 transgenic mice.

β‐amyloid protein (Aβ) deposition and hyperphosphorylation of tau are pathological characteristics of AD (1). As the role of PGI_2_ in AD development is presently unknown, we designed a study to identify the aggravating effects of PGI_2_ on AD. The major findings of this study are as follows: (i) APH‐1α/1β expression was markedly upregulated during the course of AD development; (ii) the accumulation of PGI_2_ in neuron cells induced the mRNA and protein expression of APH‐1α/1β in APP/PS1 mice; (iii) the PKA/CREB and JNK/c‐Jun signaling pathways are critical for mediating the effects of PGI_2_ on stimulating the expression of APH‐1α/1β, which is critical for γ‐cleavage of β‐APP and producing Aβ_1–42_; and (iv) Aβ_1–42_ oligomers in the CSF of APP/PS1 mice are responsible for augmenting the activity APH‐1α/1β, which potentially aggravates the pathogenesis of AD (Fig. 6).

Substantial evidence indicates that prostaglandins, such as PGE_2_ and 15d‐PGJ_2_, are important for Aβ deposition and tau tangling, which contribute to the role of COX‐2 in the pathophysiology of AD (Hoshino *et al*., 2007; Arnaud *et al*., 2009). However, knowledge concerning the specific function of PGI_2_ in the human brain is limited. Indeed, the assumption that PGI_2_ is synthesized in brain tissue *in vivo* is based on observations in primary culture of astrocytes and meningeal cells (Murphy *et al*., 1985) as well as of the expression of PGI_2_ receptor (PI) in the rodent brain (Oida *et al*., 1995; Takechi *et al*., 1996). Siegle *et al*. (Siegle *et al*., 2000) supported this hypothesis by demonstrating, via IHC and *in situ* hybridization, that glia and neuron cells in the human brain express PGI_2_ synthase (PGIS). Moreover, the expression of PGIS in the human brain was supported by the detection of 6‐keto‐PGF1α, a stable degradation product of PGI_2_, in human brain homogenates by enzyme immunoassay kits (Siegle *et al*., 2000). Our data agree with this prior work (Siegle *et al*., 2000) by demonstrating the presence of PGI_2_ in the brains of C57BL/6 mice. PGI_2_ production was elevated in the brain of APP/PS1 transgenic mice when compared with that of the WT control. PGI_2_ synthesis may result from COX‐2 upregulation in APP/PS1 transgenic mice (data not shown). Yosojima *et al*. (Yasojima *et al*., 1999) reported that COX‐2 was substantially upregulated in the affected areas of AD brains. In addition, COX‐2 is responsible for the systemic synthesis of PGI_2_ (McAdam *et al*., 1999). Therefore, PGI_2_ synthesis was markedly increased during the course of AD progression.

PGI_2_ elevation was initially found to be involved in the actions of inflammation (Ford‐Hutchinson *et al*., 1978). In addition, treatment with PGI_2_ analogs, including iloprost and treprostinil, suppressed TNF‐α expression in human myeloid dendritic cells (Kuo *et al*., 2012). Schuh *et al*. (2014) also reported that the early induction of PGI_2_ at the site of traumatic injury resulted in the aggregation of IL‐1β‐expressing macrophages as a critical reason for neuropathic pain. Although the effects of these cytokines on Aβ production are still in debating, these prior works have indicated that PGI_2_ might play its roles in AD via inducing the production of cytokines. Apart from the inflammatory effects of PGI_2_, the ability of neuron cells to express elevated amounts of PGI_2_ in APP/PS1 transgenic mice suggests that PGI_2_ may be important to the pathogenesis of AD. Because there is no report that demonstrates the functional significance of PGI_2_ in regulating Aβ deposition, we assayed for the synthesis of α‐, β‐, and γ‐secretases in PGI_2_‐treated neuronal cells. The results demonstrated that the expression of BACE‐1 and APH‐1α/1β was upregulated while ADAM‐10 expression was downregulated in PGI_2_‐treated n2a cells. Therefore, PGI_2_ elevation could possibly accelerate the deposition of Aβ_1–42_ by decreasing the expression of α‐secretase and increasing the expression of β‐ and γ‐secretases. As the roles of ADAM‐10 and BACE‐1 in Aβ deposition have been thoroughly investigated (Niemitz, 2013), we sought to determine the effects of PGI_2_ in stimulating the expression of APH‐1α/1β. Although there are no reports that demonstrate the effects of PGI_2_ in inducing the expression of APH‐1α/1β, APH‐1α/1β are required for notch pathway signaling, for γ‐secretase cleavage of β‐APP, and for Aβ protein accumulation in *C. elegans* (Francis *et al*., 2002). Indeed, APH‐1 often combines with PEN‐2, nicastrin, and PS to generate an active form of γ‐secretase complex, which is responsible for the cleavage of β‐APP and for the deposition of Aβ (De Strooper, 2003). Once APH‐1 was found in *C. elegans* (Francis *et al*., 2002), the APH‐1 complex was confirmed in several experimental models (Gu *et al*., 2003; Luo *et al*., 2003; Hansson *et al*., 2004). Along these lines, APH‐1α/1β might also be involved in regulating the deposition of Aβ_1–42_ in response to PGI_2_ stimulation.

PGI_2_ is important for regulating the expression of APH‐1α/1β, which is regulated by the PKA/CREB and JNK/c‐Jun signaling pathways and leads to Aβ_1–42_ deposition. Consistent with our observations, Su *et al*. (2003) reported that H89 treatment suppressed the production of Aβ in cells that have been stably transfected with human APP bearing a ‘Swedish mutation’. They further found that the PKA inhibitor abolishes the mature form of intracellular APP and accumulates the immature form (Su *et al*., 2003). In addition, Marambaud *et al*. (Marambaud *et al*., 1999) found that H89 inhibited the production of Aβ_1–40_ and Aβ_1–42_ in HEK293 cells that expressed the APP/PS1 genes. However, these studies were not extended to the expression of β‐ or γ‐secretases. Although the PKA inhibitor has shown similar effects in the suppression of the production of Aβ_1–42_, the role of H89 in Aβ‐induced memory deficit is not conclusively identified (Amini *et al*., 2015). In addition to the PKA signaling pathway, the JNK/c‐Jun signaling pathways have also been suggested to be involved in Aβ deposition. For example, Jung *et al*. (2015) reported that the c‐Jun N‐terminal kinase mediates the effects of auraptene on the production of Aβ by activating γ‐secretase. Shen *et al*. (2008) supported this observation by showing that JNK‐dependent activation of γ‐secretase is responsible for Aβ deposition in H_2_O_2_‐stimualted SH‐SY5Y cells. In detail, γ‐secretase as well as presenilin nicastrin is involved in mediating the effects of SP600125 on suppressing the production of Aβ_1–42_ (Kuo *et al*., 2008; Rahman *et al*., 2012). More importantly, the inhibition of c‐Jun N‐terminal kinase activation reverses the AD phenotype in APP/PS1 mice (Zhou *et al*., 2015). Along these lines, our data further found that the PKA/CREB and JNK/c‐Jun pathways are important for Aβ deposition by activating APH‐1α/1β in PGI_2_‐stimulated cells and APP/PS1 mice.

We will focus this discussion on the role of Aβ regulation in the pathogenesis of AD. Interestingly, the expression of APH‐1α/1β was upregulated when we injected (i.c.v.) the CSF of APP/PS1 mice into WT mice. This upregulation was attenuated by the addition of Aβ antibodies. These observations clearly indicate the possible role of CSF‐bound Aβ of APP/PS1 mice in upregulating the expression of APH‐1α/1β. However, previous studies have suggested that the CSF‐bound Aβ_1–42_ level progressively reduced in patients with AD (Mo *et al*., 2015). These observations indicate that the total level of Aβ_1–42_ in the CSF of APP/PS1 mice might not be critical for upregulating the expression of APH‐1α/1β. As noted by Lopez‐Gonzalez *et al*. (2015), the self‐aggregated characteristics of Aβ_1–42_ result in the Aβ_1–42_ oligomers being critical for AD initiation. In agreement with this observation, our data demonstrated that Aβ oligomer injection (i.c.v.) has the ability to stimulate the expression of APH‐1α/1β. More interestingly, NS398 blocked the effects of Aβ oligomers on inducing the expression of APH‐1α/1β. These observations clearly indicated the possible roles of Aβ oligomers in activating COX‐2, which potentially further aggravates AD. In agreement with our hypothesis, Kotilinek *et al*. (2008) also suggested that possible cross talk exists between COX‐2 and Aβ.

In conclusion, we elucidated the signaling pathway by which PGI_2_ regulates the expression of APH‐1α/1β in neuron cells of APP/PS1 mice. We found that PGI_2_ treatment upregulates the synthesis of APH‐1α/1β by activating the PKA/CREB and JNK/c‐Jun signaling pathways, which results in Aβ formation in neuronal cells. Aβ injection (i.c.v.) further stimulates the expression of APH‐1α/1β, which potentially contributes to the pathogenesis of AD.

## Experimental procedures

### Reagents

Unless otherwise specified, all reagents used for the study were described in the supporting information.

### Transgenic mice and treatments

The female wild‐type (WT) or APP/PS1 transgenic mice [B6C3‐Tg (APPswe, PSEN1dE9) 85Dbo/J (Stock Number: 004462)] were obtained from The Jackson Laboratory (Bar Harbor, ME, USA). Genotyping was performed at 3–4 weeks after birth. In selected experiments, mice at the age of 1 month were treated with NS398 (50 μg kg^−1^ day^−1^) for 5 months before determining the expression of APH‐1α/1β. Each group contains 3 mice. The brains of animals in different groups were collected after anesthesia and perfusion as previously described (Yu *et al*., 2015).

### Cerebrospinal fluid collection

Cerebral spinal fluid (CSF) was collected according to a published method (Liu *et al*., 2004) with minor modifications as previously described (Wang *et al*., 2015; Yu *et al*., 2015).

### Aβ_1–42_ preparation

The methods for preparing Aβ oligomers or fibrils had been described previously (Dahlgren *et al*., 2002; Moore *et al*., 2002; Pan *et al*., 2011). The detailed information was provided in the supportive information.

### Intracerebroventricular injection (i.c.v.)

NS398, PGI_2_, Aβ, Aβ antibody, or vehicle (PBS) were injected (i.c.v.) into WT or APP/PS1 transgenic mice as previously described (Yu *et al*., 2015). Each group contains eight mice. In selected experiments, the WT mice were injected (i.c.v.) with the CSF of APP/PS1 mice. At indicated time intervals, the brains were harvested under anesthesia and perfusion as previously described (Yu *et al*., 2015).

### Organotypic slice culture of brain tissue

Brain tissue was freshly collected from WT C57BL/6 or APP/PS1 transgenic mice at 6 months of age. Serial sections (400 μm thick) were cut using a chopper without fixation. Each group contains eight mice. The tissue sections were immediately cultured in DMEM/high‐glucose medium with 10% FBS. In a separate set of experiments, the tissues were grown in serum‐free medium for an additional 24 h before incubation with PGI_2_ (10 μm) or Aβ oligomers (1 μm) in the absence or presence of NS398 (10 μm), as previously described (Yu *et al*., 2015). The tissue sections were fixed and immunostained with APH‐1 antibody by IHC staining kit (Invitrogen, Carlsbad, CA, USA).

### Luciferase assays and live animal imaging

The live animal imaging was performed as previously described (Wang *et al*., 2015; Yu *et al*., 2015). In brief, the n2a cells that were transfected with APH‐1α/β promoter were preseeded on one side of a cerebral ventricle. PGI_2_ or vehicle (PBS) solutions were then injected (i.c.v.) into the other cerebral ventricle. At different time intervals, the mice were anesthetized and injected (i.c.v.) with luciferin at the side cerebral ventricle, which was preseeded with n2a cells. Each group contains 6 mice. The scan was performed exactly five min after luciferin introduction. All images were analyzed using Bruker *in vivo* imaging systems (MS FX PRO, Carestream, Billerica, MA, USA).

### Two‐photon imaging

*In vivo* two‐photon recording was performed as previously described (Wang *et al*., 2015; Yu *et al*., 2015). In brief, a custom‐built, two‐photon microscope that was based on a chameleon excitation laser operating at 690–1064 nm was used. The laser‐scanning unit was mounted on an upright microscope that was equipped with a water immersion objective (Zeiss; 20×, Beijing, China). The fluorescence was detected using specific antibody staining. The brain slices were stained and scanned before and after the injection (i.c.v.) of PGI_2_ or vehicle (PBS) solutions. Each group contains 6 mice.

### Quantitative real‐time PCR (qRT–PCR)

qRT–PCR assays were performed with the MiniOpticon Real‐Time PCR detection system (Bio‐Rad, Hercules, CA, USA) using total RNA and the GoTaq one‐step Real‐Time PCR kit with SYBR green (Promega, Madison, WI, USA) and the appropriate primers as previously described (Wang *et al*., 2010, 2011b,c, 2015,b). The GenBank accession number and forward and reverse primers for human or mouse BACE‐1, APH‐1α, APH‐1β, and GAPDH are provided in our previous publications (Wang *et al*., 2014c; Guan *et al*., 2015a,b; Yu *et al*., 2015). Other primers are shown in Table 2, and the gene expression values were normalized to those of GAPDH.

**Table 2:** The primer sequences for α‐, β‐, or γ‐secretases

Gene symbol	GenBank number	Sequences
ADAM‐10	NM_007399	F‐ATTGCTGCTTCGATGCCAAC R‐GCACCGCATGAAAACATCAC
PS1	NM_008943	F‐GCTTGTAGGCGCCTTTAGTG R‐CATCTGGGCATTCTGGAAGT
PS2	NM_011183	F‐AAGAACGGGCAGCTCATCTA R‐TCCAGACAGCCAGGAAGAGT
NCT	NM_021607	F‐TGTGCAGTGCCCAAATGATG R‐GGCCACATTCCAGAAAAAGGAC
PEN2	NM_025498	F‐ACTGAAAACTGCGGCATCTC R‐ATTGGGGCAGATGGGAAATG

### Immunostaining

Human SH‐SY5Y and mouse n2a cells were immunostained as previously described (Wang *et al*., 2011c). In brief, cells were permeabilized with 0.1% Triton X‐100 for 1 min at 4 °C, fixed with 4% paraformaldehyde for 10 min at 37 °C, washed with PBS (−), and incubated in buffer containing 1% BSA/PBS (−) for 10 min at room temperature. Cells were then incubated with a rabbit antibody to APH‐1 for 60 min at room temperature, washed with 1% BSA/PBS (−), and incubated in buffer containing Alexa Fluor 488‐labeled goat anti‐rabbit IgG for 60 min at room temperature. The cells were then washed five times with 1% BSA/PBS (−) before incubation in DAPI solution for five min. Finally, the cells were washed five times with 1% BSA/PBS (−) and once with deionized water before observation under confocal microscopy (Leica, TCS‐SP8, Liaoning, Shenyang, China).

### Human brain samples

Human brain samples were obtained from New York Brain Bank, serial numbers P535‐00 (normal), T4339, T4304, and 235‐95 (patients with AD). Another two normal brain samples were obtained from Fengtian Hospital of China (the patients are 59‐ and 63‐year‐old men who were diagnosed as cerebral edema, and the normal tissues are collected surrounding the tissues of cerebral edema).

The information for immunohistochemistry (IHC), cell culture, Western blot analysis, measurement of the Aβ_1–42_ or PGI_2_ concentration in the culture medium or the brain of mice, Transfection and Animal committee, was described in the supporting information.

### Statistical analysis

All data are represented as the mean ± SE of at least three independent experiments. The statistical significance of the differences between the means was determined using a Student's *t*‐test or a one‐way ANOVA, where appropriate. If the means were found to be significantly different, multiple pairwise comparisons were performed using the Tukey's test (Wang *et al*., 2015,c; Guan *et al*., 2015a,b; Yu *et al*., 2015).

## Funding

No funding information provided.

## Conflict of interest

The authors declare no competing financial interests.

## Author contributions

P. W. and P.P.G conceived and performed all of the experiments, participated in the design of the study, and wrote the manuscript. J.W.G., L.L.C., G.B.X., X.Y, and Y.W. carried out some of the experiments. P.W. and Z.Y.W. conceived the experiments, interpreted the data, and wrote the manuscript.

## Supporting information


**Data S1** Experimental procedures.
