# Supplementary material for: Prostaglandin I2 upregulates the expression of anterior pharynx‐defective‐1α and anterior pharynx‐defective‐1β in amyloid precursor protein/presenilin 1 transgenic mice
Source: Aging Cell. 2016 May 30;15(5):861–71. doi: 10.1111/acel.12495 (PMC5013024; doi:10.1111/acel.12495)
Supplement: Supplementary file 1 — Data S1 Experimental procedures. [file ACEL-15-861-s001.doc]

**Experimental Procedures**

**Reagents-** NS398, PGI_2_, Aβ_1-42_ and the inhibitors H89, SP600125 were obtained from Sigma-Aldrich Corp (St. Louis, MO, USA). Antibodies against β-actin, CREB, p-CREB (Ser 133), c-Jun, p-c-Jun (Ser 63), Aβ, Alexa Fluor 488-labeled goat anti-rabbit IgG and HRP-labeled goat anti-mouse IgG were purchased from Cell Signaling Technology, Inc. (Danvers, MA, USA). APH-1 antibody was from Merck Millipore (Bedford, MA, USA). sAPPα and sAPPβ antibodies were purchased from IBL International Corp (Toronto, ON, Canada). DAPI was obtained from Beyotime Institute of Biotechnology (Haimen, JS, China). The PGI_2_ enzyme immunoassay kits were from Bio-Swamp Life Science (Wuhan, Hubei, China). The Aβ_1-42_ enzyme immunoassay kits were from Invitrogen (Carlsbad, CA, USA). CREB, c-Jun or scramble siRNA was obtained from cell signaling technology, Inc. (Danvers, MA, USA). All reagents for the qRT-PCR and SDS-PAGE experiments were purchased from Bio-Rad Laboratories. All other reagents were from Invitrogen (Carlsbad, CA, USA) unless otherwise specified.

**Aβ_1-42_ preparation**- Freeze-drying Aβ_1-42_ protein (Stock Number: A9810, Sigma, St. Louis, MO, USA) was initially monomerized by dissolving it to a final concentration of 1 μg/μl in 100% hexafluoroisopropanal (HFIP) and the solution was aliquoted in sterile eppendorf tubes. HFIP was then evaporated under vacuum and the peptide was stored at -20℃ before reconstituent. For preparing Aβ_1-42_ oligomers, the peptide was initially resuspended in dimethylsulfoxide (DMSO) to 20 μg/μl with water bath ultrasonication for 10 min and the solution was then diluted to a final concentration of 0.2 mg/ml in phenol red-free F-12 media, and incubated at 4℃ for 24 h.

**Immunohistochemistry (IHC)-** Brain tissues were collected from WT or APP/PS1 transgenic mice at six months of age. In selected experiments, the brain tissues were collected after injection (i.c.v) of PGI_2_ (2 μg/5 μl). Serial sections (10-μm thick) were cut using a cryostat (Leica, CM1850, Germany). Slides were first rehydrated in a graded series of ethanol and then submerged in 3% hydrogen peroxide to eliminate endogenous peroxidase activity. APH-1 levels were determined using an immunohistochemical staining kit, per the manufacturer’s instructions (Invitrogen, Carlsbad, CA, USA).

**Cell culture-** Human neuroblastoma SH-SY5Y cells and mouse neuroblastoma n2a cells were grown (37 °C and 5% CO_2_) on 6 cm tissue culture dishes (10^6^ cells per dish) in the appropriate medium. In a separate set of experiments, the cells were grown in serum-free medium for an additional 24 h before incubation with NS398 or inhibitors in the absence or presence of PGI_2_.

**Western blot analysis-** Tissues or cells were lysed in radio-immune precipitation assay buffer (25 mM Tris-HCl [pH 7.6], 150 mM NaCl, 1% NP-40, 1% sodium deoxycholate, and 0.1% SDS) containing protease inhibitor cocktail (Pierce Chemical Company). The protein content of the cell lysates was determined using a bicinchoninic acid (BCA) protein assay reagent (Pierce Chemical Company). The total cell lysates (4 μg) were separated using an SDS-PAGE gel, transferred to a membrane, and probed with a panel of specific antibodies. Each membrane was only probed with one antibody. β-actin was used as a loading control. All western hybridizations were performed at least in triplicate using a different cell preparation each time.

**Measurement of the Aβ_1-42_ or PGI_2_ concentration in the culture medium or the brain of mice-**The Aβ_1-42_ or PGI_2_ levels in the media of both control and pharmacologically treated cells or the brain of mice were determined using Aβ_1-42_ or PGI_2_ enzyme immunoassay kits following the manufacturer's instructions. The total protein in the medium was used as a loading control, and the results are expressed as pg Aβ_1-42_ or pmol PGI_2_ per mg of total protein.

**Transfection-** Cells were transfected with 100 nM of a CREB or c-Jun-specific siRNA oligonucleotide. In control experiments, the cells were transfected with 100 nM of scrambled siRNA.

**Animal committee-** All animals were handled according to the care and use of medical laboratory animals (Ministry of Health, Peoples Republic of China, 1998) and all experimental protocols were approved by the Laboratory Ethics Committees of College of Life and Health Sciences of Northeastern University.
